# Case Report: Tirzepatide improves glycemic control in Rabson–Mendenhall syndrome

**DOI:** 10.3389/fendo.2026.1945251

**Published:** 2026-08-28

**Authors:** David Araújo-Vilar, Amaia Vela, Ana I. Castro, Antía Fernández-Pombo, Teresa Prado-Moraña, Everardo J. Díaz-López, José Antonio Sánchez-Aparicio, Rosa Martínez, Luis Castaño, Sofía Sánchez-Iglesias

**Affiliations:** 1 UETeM-Molecular Pathology Group, Department of Psychiatry, Radiology, Public Health, Nursing and Medicine, IDIS-CIMUS, University of Santiago de Compostela, Santiago de Compostela, Spain; 2 Division of Endocrinology and Nutrition, University Clinical Hospital of Santiago de Compostela, Santiago de Compostela, Spain; 3 Department of Pediatrics, EHU, Barakaldo, Spain; 4 Division of Pediatric Endocrinology, Hospital Universitario Cruces, Barakaldo, Vizcaya, Spain CIDERER, CIBERDEM, Biobizkaia, Barakaldo, Spain; 5 CIBER Fisiopatología de la Obesidad y la Nutrición (CIBERobn), Madrid, Spain; 6 Division of Endocrinology and Nutrition, University Clinical Hospital of Ourense, Ourense, Spain; 7 Department of Ophthalmology, Biocruces Bizkaia Health Research Institute, Cruces University Hospital, University of the Basque Country, Barakaldo, Spain; 8 Endocrinology and Diabetes Research Group, Biobizkaia Health Research Institute, Barakaldo, Vizcaya, Spain

**Keywords:** diabetes mellitus, insulin receptor, insulin resistance, Rabson – Mendenhall syndrome, tirzepatide

## Abstract

**Background:**

Rabson–Mendenhall syndrome (RMS) is an extremely rare autosomal recessive disorder caused by variants in the INSR gene, which encodes the insulin receptor. The condition is characterized by profound insulin resistance, resulting in diabetes that is exceptionally difficult to control and carrying a poor prognosis, typically in the second to third decade of life. Death most commonly occurs due to ketoacidosis and severe infections. There is no established consensus on optimal treatment; initial therapy usually includes metformin and pioglitazone, often combined with SGLT2 inhibitors, followed by maintenance with high doses of insulin.

**Case presentation:**

Two patients with genetically confirmed RMS were treated with subcutaneous tirzepatide for 3 months (2.5–3.3 mg weekly), followed by a 3-month washout period. Clinical, biochemical, and body composition parameters were assessed at baseline, after treatment, and after withdrawal. After 3 months of treatment, HbA1c decreased from 8.9% to 6.3% in one patient and from 10.3% to 8.1% in the other. Insulin resistance improved in one case, whereas insulinemia decreased markedly in the other. Glycemic control deteriorated after treatment withdrawal. Tirzepatide was generally well tolerated, although both patients experienced a reduction in fat mass, and one developed proliferative retinopathy, probably related to the rapid fall in HbA1c.

**Conclusions:**

Low-dose tirzepatide improved glycemic control in RMS and may represent a potential adjunctive therapy, warranting careful monitoring.

## Introduction

Pathogenic variants in the gene encoding the insulin receptor (*INSR*) are associated with clinical conditions characterized by severe insulin resistance (1). These include leprechaunism or Donohue syndrome (2, 3), Rabson–Mendenhall syndrome (RMS) (4), type A insulin resistance syndrome (5) and intermediate or atypical forms of severe insulin resistance, often with cutaneous features (acanthosis, hypertrichosis), endocrine abnormalities (hyperandrogenism), and impaired growth (1). These conditions could be regarded as manifestations of varying severity of the same disorder, with leprechaunism being the most severe and type A insulin resistance syndrome the mildest (2, 5, 6). Disease severity, which influences life expectancy, appears to correlate with the type of *INSR* variant, such that greater loss of insulin receptor function results in more severe disease (2, 6–8).

The clinical features of RMS, an autosomal recessive disorder, usually appear in early childhood and include growth retardation, facial dysmorphism, dental anomalies, gingival hyperplasia, severe acanthosis nigricans, pineal hyperplasia, genital enlargement, precocious puberty or multiple ovarian cysts, and frequent renal complications (4, 9). From an early age, affected children show markedly elevated plasma insulin levels; fasting hypoglycemia may occur, and diabetes typically develops early in life (4).

There is no consensus on the initial therapeutic approach for glycemic control in RMS. Metformin and pioglitazone are considered first-line treatments; however, their efficacy is limited and tends to wane over time (10), necessitating combinations with novel agents such as sodium–glucose cotransporter 2 inhibitors (SGLT2i) (11, 12). Exogenous insulin is typically required at extremely high doses, but frequently fails to achieve adequate glycemic control due to the primary receptor defect (11). Metreleptin has shown sustained glycated hemoglobin (HbA1c) reduction over long-term follow-up, associated with BMI decrease (13). Finally, recombinant human Insulin-like Growth Factor 1 (mecasermine) may transiently improve glycemic control in select cases, but heterogeneous responses, short half-life, and management complexity limit its utility (8). Survival typically extends into the second decade of life; mortality is most commonly attributable to acute and chronic complications of severe diabetes, particularly recurrent ketoacidosis and infections (11).

Recently, two studies (14, 15) have shown that the dual GIP/GLP-1 receptor agonist tirzepatide achieves good metabolic control in generalized lipodystrophies. Given that these patients exhibit severe insulin resistance despite leanness, we used tirzepatide in two patients with RMS and poor metabolic control, achieving a good response at a weekly dose of 2.5–3.3 mg.

## Case presentation

This study was approved by the Clinical Research Ethics Committee of the Xunta de Galicia (code: 2022/383). The study was conducted in accordance with the Declaration of Helsinki, and written informed consent was obtained from the patient or their legal guardians.

Subject #1: A 22-year-old male diagnosed with RMS at age 6, due to compound heterozygous *INSR* variants c.2740C>T, p.(Arg914Cys) and c.2776C>T, p.(Arg926Trp). Growth was normal, but he had dental crowding, gingival hypertrophy, and severe acanthosis nigricans, with persistently elevated insulin and C-peptide concentrations. Baseline clinical and biochemical parameters are shown in **Table 1**. Diabetes developed at 13 years and remained well controlled until 17 on metformin, pioglitazone, and canagliflozin. Thereafter, despite this regimen, metabolic control deteriorated (HbA1c: 8.9%), leading to off-label tirzepatide initiation at age 22. No macroangiopathic complications of diabetes were present, and he had only mild microalbuminuria but not diabetic retinopathy prior to tirzepatide.

Subject #2. A 14-year-old girl with RMS diagnosed at 11 months due to compound heterozygous *INSR* variants c.425G>A, p.(Gly142Asp) and c.877T>C, p.(Cys293Arg). Clinical features include growth retardation, coarse facies, dental crowding, premature thelarche, acral skin redundancy, ovarian torsion secondary to cysts, primary amenorrhea, clitoromegaly, and severe acanthosis nigricans, with persistently elevated insulin and C-peptide. Baseline data are shown in **Table 1**.

**Table 1:** Demographic, anthropometric, and biochemical characteristics of subjects #1 and #2 at baseline and in response to tirzepatide treatment.

Demographic, anthropometric, and biochemical characteristics	Subject #1 (male)					Tirzepatide (6 months)	%△	Subject #2 (female)				
	Baseline	Tirzepatide (3 months)	%△	No tirzepatide (3 months)	%△			Baseline	Tirzepatide (3 months)	%△	No tirzepatide (3 months)	%△
Age (y.)	22	22		22		23		14	14		14	
Weight (kg)	58,9	56.4	-4.2	60	6.4	56.7	-5.5	28.6	26.6	-6.6	31	16
Height (cm)	175	175	0	175	0	175	0	133	133	0	133	0
BMI (kg/m^2^)	19.2	18.6	-3.1	19.6	5.4	18.5	-5.6	16.2	14.8	-8.6	17.5	18.2
Total fat (kg)	9.3	7.8	-16	10.2	31	8.2	-19.6	7.42	5.5	-25.9	NA	—
Total fat (%)	15.9	13.6	-14.5	17.0	25	14.5	-14.7	26	20.7	-20.4	NA	—
Lean mass (kg)	46.9	46.5	-0.9	47.2	1.5	45.8	-2.3	20.1	20.2	0.5	NA	—
Lean mass (%)	79.6	82.4	3.5	78.7	-4.5	81.2	3.2	70.3	75.9	8	NA	—
A1c (mmol/mol)	74	45	-39	81	80	60	-26	89	65	-27	84	29
A1c (%)	8.9	6.3	-29	9.6	52	7.6	-20	10.3	8.1	-21	9.8	21
Insulin (pmol/L)	1410	894	-37	2580	189	1224	-53	45900	6864	-85	3510	-49
C-peptide (pmol/L)	1225	1258	2.7	2549	103	1099	-57	6620	7779	18	7646	-1.7
HOMA-IR	73.2	27.9	-62	238	753	62.9	-74	NA	NA	—	NA	—
Leptin (ng/mL)	<1	<1	—	1	—	<1	—	<1	<1	—	NA	—
Adiponectin (µg/mL)	6.3	7.98	26.7	9.4	18	17	81	>30	>30	—	NA	—
eGFR (mL/min/1.73 m²)	136	139	2	141	1.4	133	-5.7	189	141	-25	172	22
AST (U/L)	16	13	-19	14	8	14	0	19	31	63	24	-23
ALT (U/L)	18	10	-44	20	100	10	-50	14	33	136	22	-33
Total cholesterol (mmol/L)	4.03	3.28	-19	3.90	19	3.3	-15	4.75	5.63	19	5.92	5
Triglycerides (mmol/L)	0.64	0.37	-42	0.62	68	0.54	-13	0.89	0.79	-11	0.85	8
LDLc (mmol/L)	2.61	2.07	-21	2.38	15	2.0	-16	2.66	2.66	0	3.13	18
HDLc (mmol/L)	1.06	1.03	-2.8	1.16	13	1.0	-14	1.68	2.61	55	2.40	-8
IGF1 (ng/mL)	267	251	-6	282	12	231	-18	<15	<15	—	<15	—
UACR (mg/mmol)	3.8	5.3	39	2.6	-51	2.5	-4	5.3	3.1	-42	NA	—

BMI, body mass index; HbA1c, glycated hemoglobin; HOMA-IR, homeostatic model assessment for insulin resistance; eGFR, estimated glomerular filtration rate; AST, aspartate aminotransferase; ALT, alanine aminotransferase; LDLc, low-density lipoprotein cholesterol; HDLc, high-density lipoprotein cholesterol; IGF1, insulin-like growth factor 1; UACR, Urine Albumin-to-Creatinine Ratio; NA, not available.

In subject#2, the HOMA-IR index was not calculated as she was receiving exogenous insulin treatment.

Diabetes onset at 3.7 years; initially treated with metformin (3 years), then metreleptin (~6 years). Insulin U-500 (200 IU QD) was initiated at 10.5 years and pioglitazone at 13.3 years, without adequate metabolic control. In April 2025, she developed diabetic ketoacidosis (HbA1c of 13.5%) with poor dietary adherence. After discharge, HbA1c improved to 10.3%, leading to consideration of off-label tirzepatide. As in Subject #1, no macroangiopathy was present; mild microalbuminuria but no retinopathy was documented pre-treatment.

## Experimental design

During the week prior to the initiation of tirzepatide treatment, both patients underwent a general physical examination. Daily energy intake was estimated from patient-reported dietary intake. Fat mass and fat-free mass were determined using dual-energy X-ray absorptiometry (DXA) with a Lunar DPX model (GE Healthcare Lunar; Madison, WI). Blood samples were collected following a 12-hour overnight fasting period. Standardized methods were employed by local laboratories in each center for measuring HbA1c, liver enzymes, triglycerides and total and fractionated cholesterol levels, insulin, C-peptide, leptin, adiponectin, creatinine, IGF-1, and urinary albumin/creatinine ratio, with rigorous quality control and assurance procedures in place.

The treatment protocol consisted of subcutaneous administration of tirzepatide once weekly using a cautious dose-titration schedule. As there is no established tirzepatide dosing regimen for Rabson–Mendenhall syndrome, and considering the low or low-normal BMI of both patients, treatment was started at a dose lower rather than initiating treatment at the standard labelled starting dose. A 10-mg multidose pen was used. The initial administration consisted of 10 clicks, corresponding to an estimated dose of approximately 1.7 mg. As this dose was well tolerated in both patients, the dose was increased to 15 clicks (approximately 2.5 mg) in the second week. Further dose adjustments were made in 5-click steps, corresponding to estimated increments of approximately 0.8 mg, according to gastrointestinal tolerance, weight loss and glycemic control, assessed by continuous glucose monitoring (FreeStyle Libre, Abbott Laboratories, Abbott Park, Illinois, USA). Treatment was continued for three months. The dose finally maintained was 2.5 mg once weekly in Subject #1 and 3.3 mg once weekly in Subject #2. Higher doses were not maintained because of gastrointestinal discomfort and because satisfactory improvement in glycemic control had already been obtained at these doses. Following a three-month washout period without tirzepatide, the subjects were reassessed once again. In subject #1, tirzepatide was reintroduced after the washout period at a weekly dose of 2.5 mg, and the patient was reassessed at 3 and 6 months.

## Results

**Figure 1:**
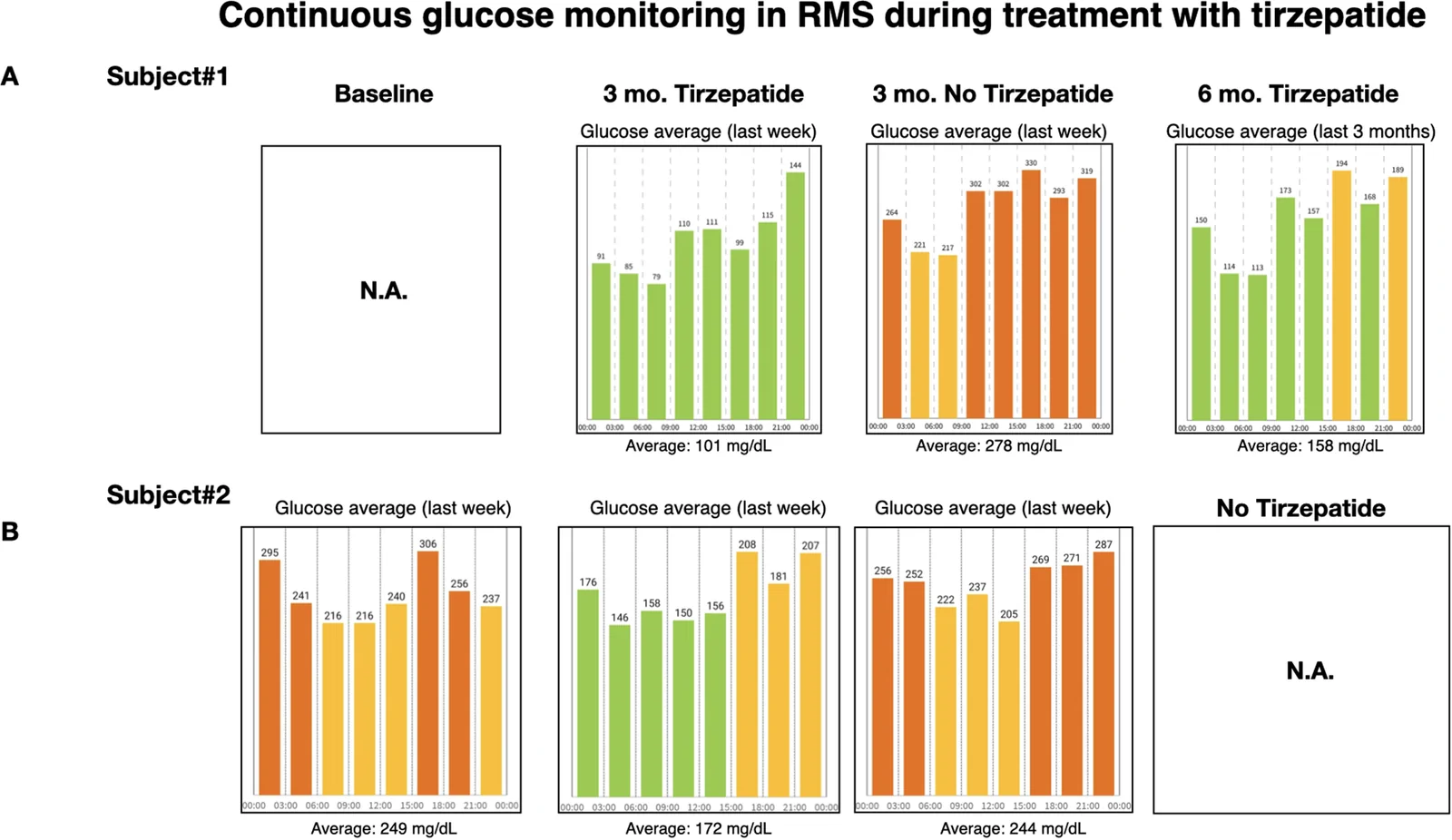
**(A)** Subject #1 and **(B)** subject #2. Representative continuous glucose monitoring profiles obtained at baseline, after 3 months of tirzepatide treatment, and after a 3-month washout period. Additional recordings obtained after tirzepatide reintroduction in Subject #1 are also shown. Treatment was associated with a substantial reduction in hyperglycaemia and glycaemic variability, whereas withdrawal resulted in deterioration of glucose control. The reproducible improvement observed after reintroduction of tirzepatide in Subject #1 provides additional support for a treatment effect in this ultra-rare disorder characterized by extreme insulin resistance. N.A., Not available.

Subject #1. After 3 months of tirzepatide (2.5 mg weekly), metabolic control improved (HbA1c decreased from 8.9% to 6.3%) with a 62% reduction in insulin resistance (HOMA-IR; **Table 1**). Treatment was well tolerated, with mild nausea and gastric discomfort, reduced appetite, and 4.2% weight loss (fat mass −3.1%) under regular physical activity. Based on the self-reported diet, a daily reduction in intake of approximately 250 kcal was observed. No hypoglycemia occurred. After discontinuation, metabolic control worsened at 3 months (HbA1c 9.6%), with increased insulin resistance and fat mass recovery (**Table 1**). Tirzepatide was reintroduced after the washout period at a weekly dose of 2.5 mg. At 3 months, a decrease in HbA1c to 7.8% was observed, and at 6 months HbA1c was 7.6%. By the end of this period, a weight loss of 3.3 kg was documented, mainly at the expense of fat mass (**Table 1**), together with an improvement in insulin resistance (HOMA-IR showed a 74% reduction). An example of the continuous glucose monitoring profiles is shown in **Figure 1A**. At 10 months, fundus photography showed no diabetic retinopathy.

Subject #2. After 3 months of tirzepatide (3.3 mg weekly), metabolic control improved (HbA1c decreased from 10.3% to 8.1%) with an 85% reduction in insulinemia (**Table 1**). Treatment was well tolerated. Weight decreased by 2 kg (-6.6%), entirely from fat mass (−25.9%), with low–moderate physical activity. Based on the self-reported diet, a daily reduction in intake of approximately 180–200 kcal was observed. No hypoglycemia occurred. After discontinuation, metabolic control worsened at 3 months (HbA1c 9.8%) with a 4.4 kg weight gain. An example of the continuous glucose monitoring profiles is shown in **Figure 1B**. Three months after tirzepatide discontinuation, the patient presented with acute bilateral vision loss due to proliferative diabetic retinopathy with severe macular edema; ophthalmologic examination 6 months earlier was normal. Treatment included intravitreal dexamethasone implants, intravitreal aflibercept, and bilateral panretinal photocoagulation; one eye required posterior vitrectomy for tractional disease. These interventions prevented progression to blindness, but ongoing treatment and follow-up are required.

## Discussion

RMS is a disorder characterized by severe insulin resistance and carries a dismal prognosis, generally related to diabetic ketoacidosis (11). As previously discussed, no curative treatment exists for this condition, and therapy aims to achieve acceptable metabolic control, typically with insulin sensitizers and high-dose insulin (10, 11). The addition of SGLT2i may contribute to improved metabolic control (11, 12), whereas metreleptin or mecasermin remain drugs with restricted use (8, 13).

Low-dose tirzepatide improved metabolic control in two RMS patients and was reasonably well tolerated. Subject #1 achieved target HbA1c within 3 months, with marked deterioration after discontinuation; weight loss (<5%) was modest and fat mass–driven. Subject #2 showed a smaller HbA1c reduction without reaching targets; dose escalation was limited by gastrointestinal intolerance and excessive fat mass loss (-25.9%). Nevertheless, the evident worsening of metabolic control upon drug discontinuation supports its utility in this condition.

An additional observation supporting the efficacy of tirzepatide in RMS is the reproducible pattern observed following treatment withdrawal and reintroduction. Both subjects experienced deterioration in glycemic control after discontinuation, while Subject #1 showed renewed improvement after tirzepatide was restarted. Although uncontrolled observations should be interpreted with caution, this within-subject response pattern strengthens the likelihood of a genuine treatment effect in a condition for which randomized clinical trials are unlikely to be feasible.

Although reduced caloric intake and weight loss undoubtedly contributed to the metabolic improvements observed in our patients, several findings suggest that additional mechanisms may be involved. The magnitude of HbA1c reduction, particularly in Subject #1, appears disproportionate to the relatively modest weight loss achieved. Moreover, the marked deterioration in glycemic control observed after tirzepatide withdrawal, together with the subsequent improvement following reintroduction in Subject #1, provide a compelling within-subject signal supporting a sustained treatment effect. Experimental and clinical studies have shown that tirzepatide improves insulin sensitivity beyond what would be predicted by weight loss alone, with a greater improvement in insulin sensitivity per unit of weight loss than that observed with semaglutide (16). Although the precise mechanisms remain unclear, these observations raise the possibility that dual GIP/GLP-1 receptor agonism may have therapeutic potential in severe genetic insulin resistance syndromes beyond its effects on appetite and body weight.

The poorer response in Subject #2 could be attributed to worse baseline metabolic control, partly conditioned by the type of variants affecting the α-subunit of the insulin receptor (17). In addition, extremely low circulating IGF-1 levels may have contributed to the less favorable response, given the potential compensatory role of IGF signaling in severe insulin receptor dysfunction (18).

A noteworthy observation was the late development of proliferative diabetic retinopathy in subject #2, occurring 3 months after tirzepatide discontinuation. Rapid HbA1c reductions have been associated with onset or worsening of diabetic retinopathy (19); a similar phenomenon has been reported with semaglutide, though evidence remains limited and inconsistent (20). Although causality cannot be established in a single case, the occurrence of proliferative diabetic retinopathy after treatment discontinuation and following a marked reduction in HbA1c is more consistent with the well-described phenomenon of early worsening of diabetic retinopathy associated with rapid glycemic improvement than with a direct adverse effect of tirzepatide.

Treatment at the indicated doses was well tolerated, apart from occasional gastrointestinal discomfort.

An obvious limitation of this study is the small sample size and short treatment period. In addition, fractional dosing was obtained by partial advancement of the dose selector of a multidose pen, a procedure not validated by the manufacturer; therefore, the reported doses should be considered approximate. We are aware that these limitations affect result interpretation, but this is an ultra-rare condition with limited therapeutic alternatives, representing a proof-of-concept. Nevertheless, the deterioration after drug discontinuation supports its beneficial effect.

In summary, low-dose tirzepatide could be considered a therapeutic option to achieve better metabolic control in RMS, alongside standard therapy, but with close monitoring for weight loss and development of diabetic retinopathy.

## Patient perspective

Both patients reported that they perceived a clear improvement in their glycemic control during tirzepatide treatment and were willing to continue treatment. They considered the doses finally maintained (2.5 mg/week in Subject #1 and 3.3 mg/week in Subject #2) acceptable. Gastrointestinal discomfort at higher doses was unpleasant for both patients, although no vomiting or diarrhea occurred. Overall, both patients considered that the improvement in glycemic control obtained with treatment was clinically relevant for them.

## Data Availability

The genetic variants and relevant clinical data are reported in the manuscript. As this is a two-patient case series, individual-level data are not deposited in a public repository because public disclosure could compromise participant anonymity. Additional anonymized data may be made available by the corresponding author upon reasonable request and subject to ethical approval and applicable data-protection requirements.
